# Synthesis of Holey Graphene Oxide and Its Application as an Adsorbent for Heavy Metal Removal from Aqueous Solutions

**DOI:** 10.1021/acsomega.5c00598

**Published:** 2025-06-25

**Authors:** Gizem Kahve Yıldırım, Yavuz Sürme, Mustafa Uçan, İbrahim Narin

**Affiliations:** † Faculty of Sciences, Department of Chemistry, 52989 Nigde Omer Halisdemir University, 51240 Niğde, Turkiye; ‡ Industrial Raw Materials and Construction Materials Application and Research Center, Nigde Omer Halisdemir University, 51240 Niğde, Turkiye; § Faculty of Pharmacy, Basic Pharmacy Sciences, 52958 Erciyes University, 38280 Kayseri, Turkiye

## Abstract

Improving advanced materials for the removal of heavy metal ions in aquatic systems is an urgent need because the contamination of waters by heavy metals causes significant environmental toxicity. In this study, holey graphene oxide (hGO), an advanced version of graphene oxide, was synthesized and its potential use as an adsorbent in a dispersive solid phase extraction (d-SPE) technique was evaluated. The characterization of hGO was achieved by Fourier-transform infrared spectroscopy, X-ray diffraction, field emission digital scanning electron microscope, Brunauer–Emmett–Teller, and energy-dispersive X-ray spectroscopy-mapping techniques. The adsorption performance of hGO on the simultaneous removal of Pb (II), Ni (II), Cd (II), Co (II), and Cu (II) metal ions was evaluated by the d-SPE method, and the optimization results revealed that quantitative removal of heavy metal ions was achieved at pH 4 for 200 s of equilibrium by using 50 mg of adsorbent. The results demonstrated that hGO exhibited competitive performance, achieving removal efficiencies of 97.8% [Pb (II)], 94.2% [Ni (II)], 97.4% [Cd (II)], 96.5% [Co (II)], and 91.6% [Cu (II)] at optimum conditions for targeted heavy metals. The optimized d-SPE method was applied to remove the heavy metal ions in certified reference material and real water samples. The hGO showed potential as an efficient adsorbent for water remediation from heavy metal pollution.

## Graphical abstract

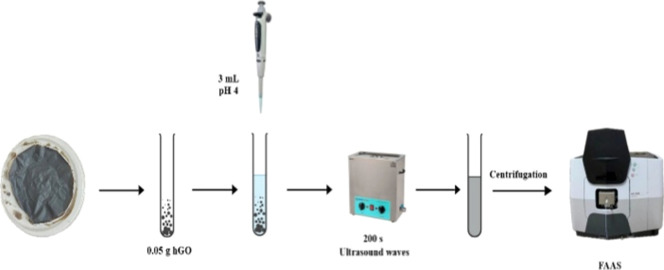

## Introduction

Holey graphene oxide (hGO) is a nanomaterial with nanoscale holes obtained by hydrothermal etching of graphene oxide (GO), and these pores are very effective in increasing the surface area.^1,2^ In this way, the gap between the GO layers is reduced, allowing more ions to adhere.^3^ Although the hGO is a new material, it has attracted widespread attention on the latest research as an effective material used to increase mass transport in application areas such as water and air purification, gas purification and gas storage, and catalyst and electrochemical energy storage.^1,4^ Because the hGO has the advantages such as hydrophilicity, presence of functional groups (which enable strong interactions with heavy metal ions) such as hydroxyl, epoxy, and carboxyl groups,^5^ and porous structure,^6^ it is possibly the perfect candidate for a new adsorbent^7,8^ for solid-phase extraction.

Environmental contamination by heavy metals is a critical issue due to their nonbiodegradable nature, persistence in the environment, and toxic effects on living organisms.^9,10^ Industrial activities, including mining, electroplating, and waste disposal, contribute significantly to the release of heavy metals such as lead (Pb), cadmium (Cd), etc., into water sources, posing severe health risks to human populations and aquatic ecosystems.^11,12^ Therefore, there is a pressing need for effective materials capable of removing heavy metals from aqueous solutions.

The dispersive solid-phase extraction (d-SPE) method is based on the ultrasound-assisted dispersion of the solid sorbent in the matrix solution, which is a modified version of classical SPE. The d-SPE technique reduces the process duration, cost, usage of solvents, operational steps, and the need for matrix removal.^13^

This research aims to provide a comprehensive understanding of the synthesis and characterization of hGO and its adsorption behavior after the simultaneous removal of Pb (II), Ni (II), Cd (II), Co (II), and Cu (II) metal ions in aqueous solution by the d-SPE technique.

## Experimental Section

### Chemicals and Reagents

The chemical compounds and stock reagents used in the experiments were of analytical grade and obtained from Sigma (Sigma-Aldrich, Munich, Germany) and Merck (Merck, Darmstadt, Germany). Stock and standard metal solutions were prepared from nitrate salts by dissolving them in doubly distilled water. Model test solutions were prepared by diluting stock metal solutions. Binary buffer solutions (H_3_PO_4_//NaH_2_PO_4_ for pH 2–3, HAc//NaAc for pH 4–5, and NaH_2_PO_4_//Na_2_HPO_4_ for 6–8) were used to adjust the pH of the model solutions.

### Synthesis of hGO Adsorbent

The GO was first synthesized using the Hummers method^14^ prior to using it in the synthesis of hGO. A solution was prepared by adding 50 mL of distilled water to 0.10 g of synthesized GO. Then, 5 mL of H_2_O_2_ was added to obtain a mixture. This mixture was boiled in a controlled manner at approximately 100 °C for 4 h. After the mixture reached room temperature, it was centrifuged to obtain hGO. The resulting hGO was cleaned by deionized water to remove excess H_2_O_2_. The obtained hGO was dried in an oven at 40 °C for 24 h prior to use in d-SPE experiments.^15,16^

### Instrumentation

The characterization of the synthesized hGO was conducted using several techniques to determine its surface, structural, and adsorption properties. The surface morphologies and adsorption characteristics of the hGO adsorbent were investigated by using a Zeiss (Wetzlar, Germany) Gemini 500 model field emission digital scanning electron microscope (FESEM)–energy-dispersive X-ray spectroscopy (EDS). The X-ray diffraction (XRD) measurements were conducted by using a Panalytical (United Kingdom) Empyrean model X-ray diffractometer. Fourier-transform infrared spectroscopy (FTIR) spectra were obtained by a Bruker (MA, USA) Vertex 70 model Fourier transform infrared spectrometer. The surface area and surface porosity of hGO were analyzed by using a Micromeritics model Gemini VII Brunauer–Emmett–Teller (BET) analyzer. The atomic absorbances of heavy metal ions were measured by using an air-acetylene flame equipped Shimadzu AA 7000 flame atomic absorption spectrometer (Kyoto, Japan). The aqueous phase and adsorbent were separated from each other using a Nuve NF 400 (Ankara, Türkiye) model centrifuge. The pH values of the model solutions were measured using a Hanna (RI, USA) HI 2211 pH meter. In the d-SPE method, an Alex Machine AXUY-06 model ultrasonic bath (Istanbul, Turkey) was used as the ultrasound source in order to ensure a fast and effective interaction between the hGO adsorbent and heavy metal ions.

### d-SPE Procedure

50 mg of hGO was weighed and added into a conical bottom polyethylene tube, and 3 mL of pH 4 buffer solution was added to it. Then, 2 μg of Cd (II), 10 μg of Cu (II), and 20 μg of Pb (II), Ni (II), and Co (II) containing stock multimetal solutions were added to the test tube. The final volume of the solution was completed to 10 mL by using deionized water. The obtained model solution was sonicated by placing it in an ultrasonic bath for 200 s. After sonication, the solution was centrifuged to separate the adsorbent and solution. The concentrations of heavy metal ions Pb (II), Ni (II), Cd (II), Co (II), and Cu (II) were determined using FAAS.^17^

## Results and Discussion

### Characterization of the hGO Adsorbent

Chemical characteristics of GO and hGO were investigated by FTIR and XRD, and morphological structures were analyzed by FESEM techniques. The adsorbed metal ions were analyzed by EDS and EDS-mapping techniques. The FTIR results are given in Figure 1.

**Figure 1:**
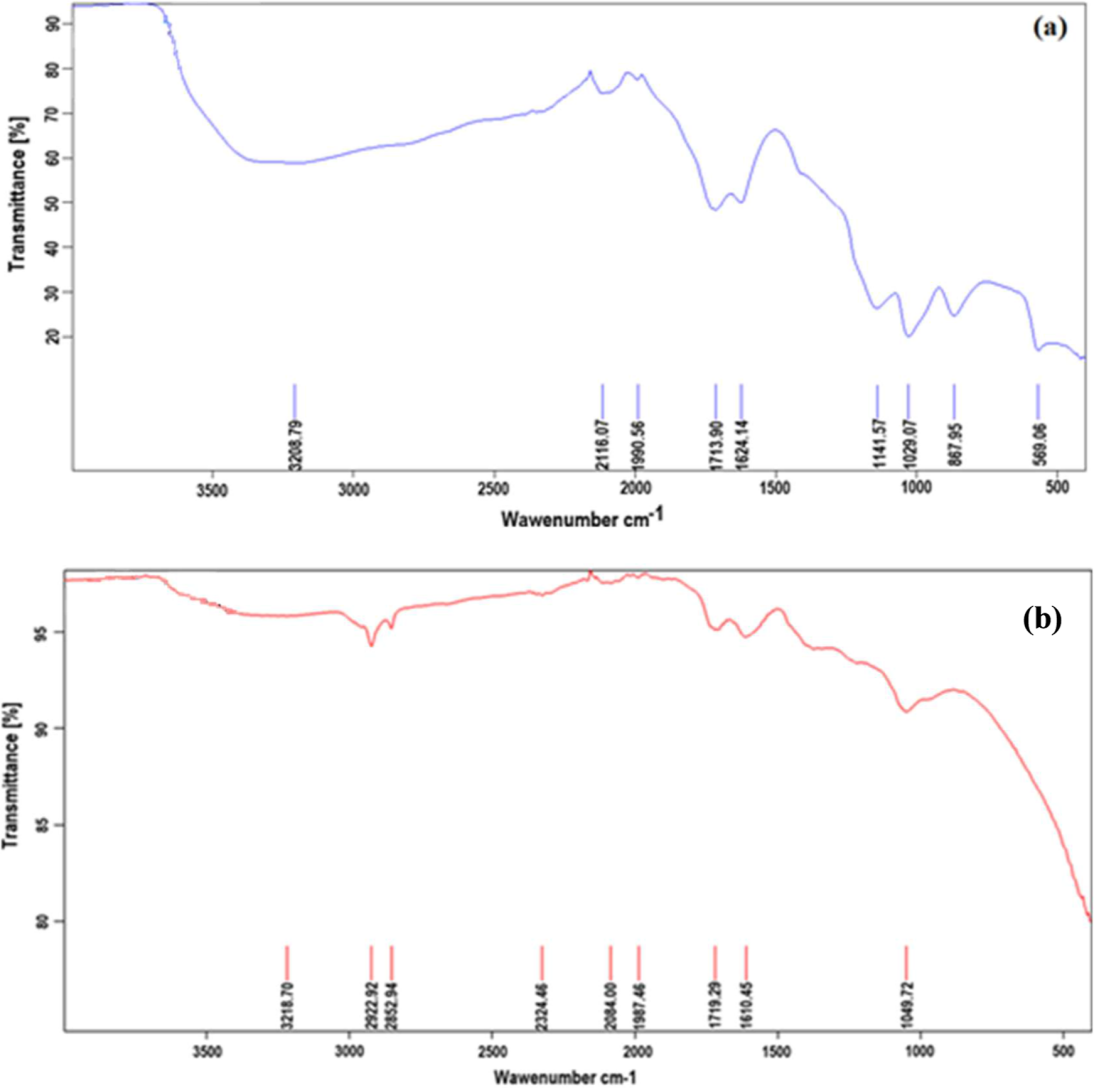
FTIR spectra of GO (a) and hGO (b).

The FTIR spectrum of GO in Figure 1a shows a broad peak around 3200 cm^–1^ due to the stretching vibrations of O–H bonds. The peak appearing at 1719 cm^–1^ is typically associated with C=O stretching vibrations of the carboxyl group on the GO surface. The peak appearing at 1624 cm^–1^ could be corresponded to C=C stretching from the sp^2^ carbon atoms. The peak at 1029 cm^–1^ indicates the stretching of C–O groups. Figure 1b shows that the intensities of the peaks appeared at 3200, 1719, and 1049 cm^–1^ are lower, indicating a partial reduction of hydroxyl, carbonyl, and carboxyl groups due to the structural changes from hole formation.^18–20^

The transformation from GO to hGO, where the latter has partially reduced functional groups, results in a more porous structure, which enhances its applicability in adsorption processes.

The XRD analysis of GO (2-1) and hGO (2-2) was also carried out, and the obtained results are given in Figure 2.

**Figure 2:**
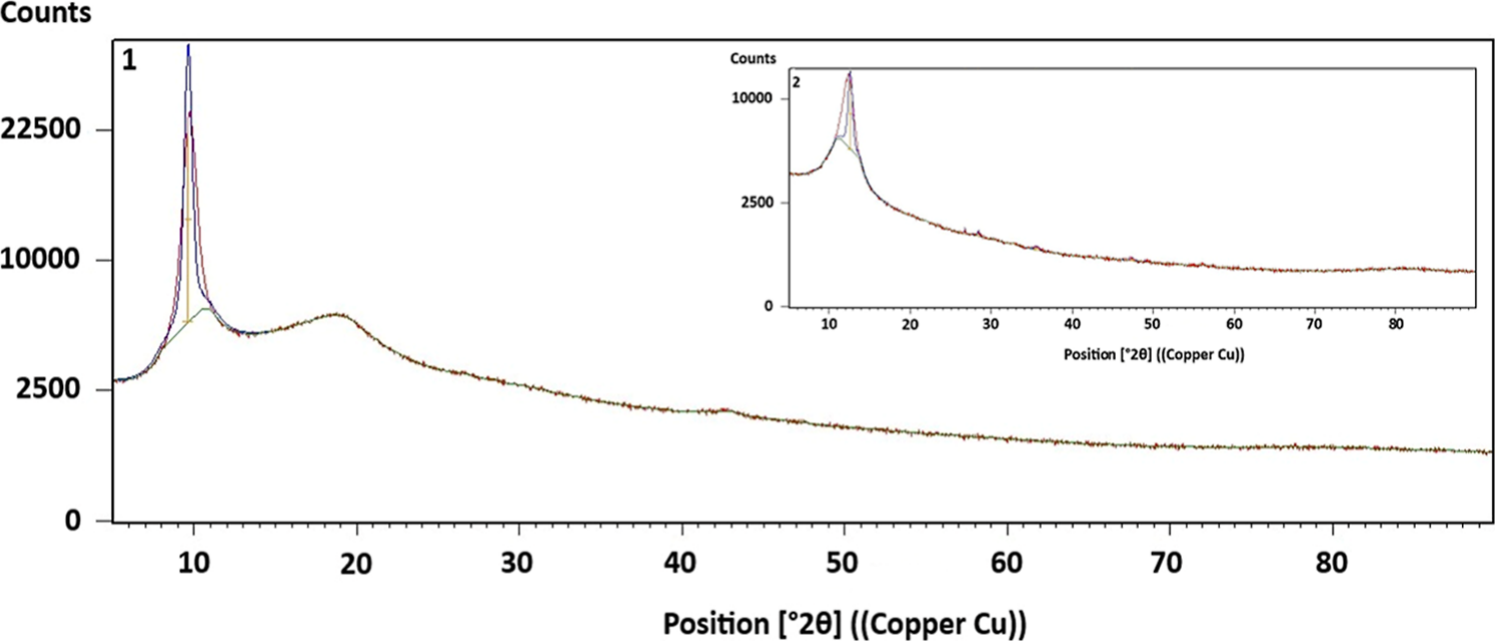
XRD patterns of GO and hGO.

GO shows a distinct XRD pattern, which reflects its structural characteristics primarily around **2θ** = **10.12°** to **11.6°** ^21^ after oxidation of graphite. According to Figure 2-1, the peak that appeared at **2θ** = **10.15°** is a typical GO peak,^22^ and the XRD peak of hGO (Figure 2-2) was shifted to **2θ** = **11.64°** with lower intensity due to the introduction of holes and structural defects.^23^

FESEM images of GO and hGO samples in Figure 3 show the characteristic morphological features of both GO and hGO. The wrinkled and layered structure of GO is shown in Figure 3a,b, while the hole formation and fractures of hGO (Figure 3c,d) can be observed in Figure 3. These observations can be considered as successful proofs of the formation layered GO and hGO. The formation of holes in GO sheets is in accordance with the literature.^6^

**Figure 3:**
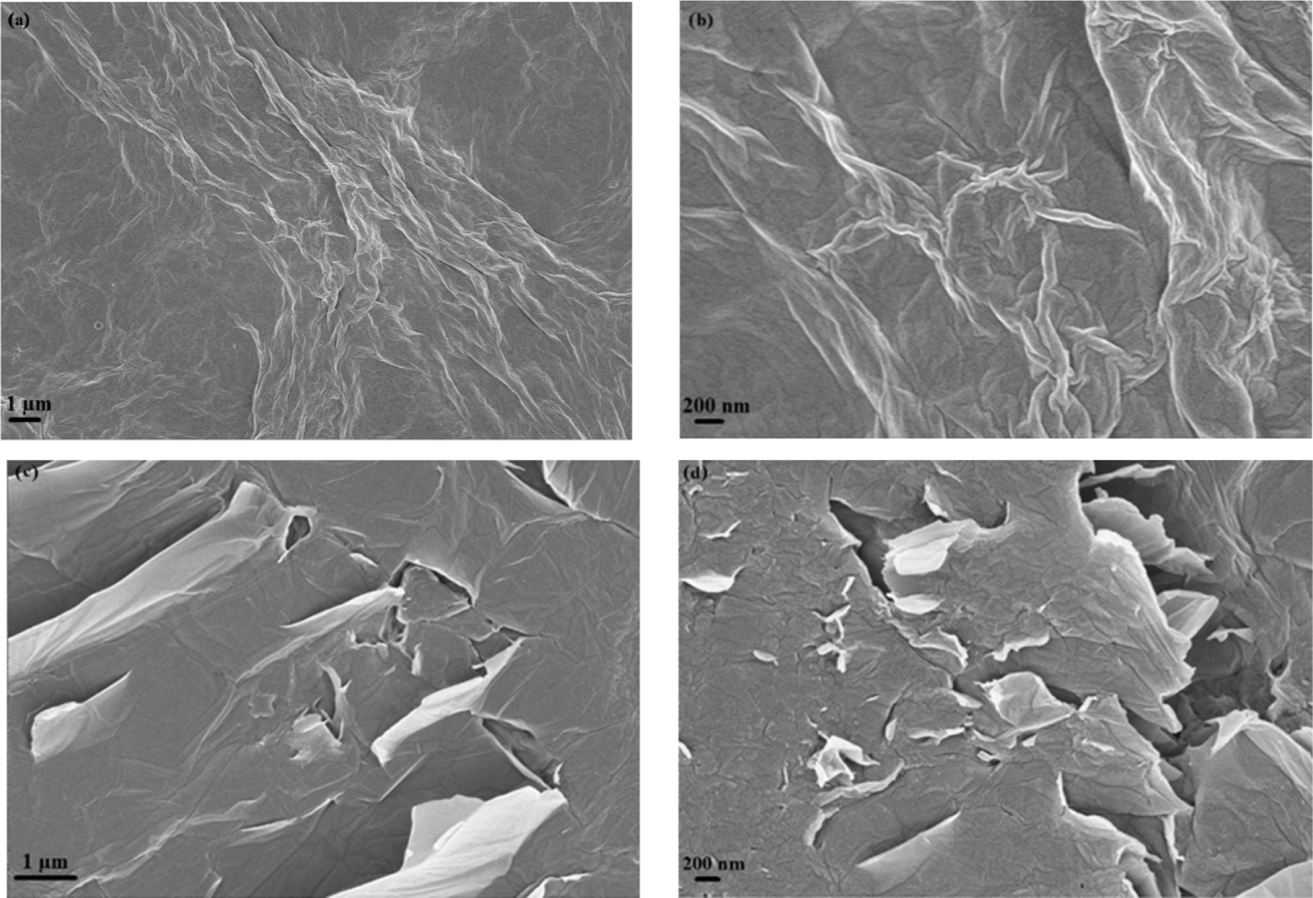
FESEM images of GO and hGO samples at different magnifications (a,b: GO; c,d: hGO).

The surface area and pore size of the GO and hGO surface were characterized by the BET technique. BET analyses illustrate that the BET surface area values of GO and hGO were 1.9667 m^2^/g and 124.1001 m^2^/g, while the single point adsorption total pore volumes of pores were 0.005789 cm^3^/g and 0.792288 cm^3^/g, respectively. These results clearly indicate that hGO has a larger surface area where the metal ions could easily be adsorbed.

Figure 4 shows the EDS spectrum, and Figure 5 exhibits the mapping analysis of the hGO samples with adsorbed heavy metals.

**Figure 4:**
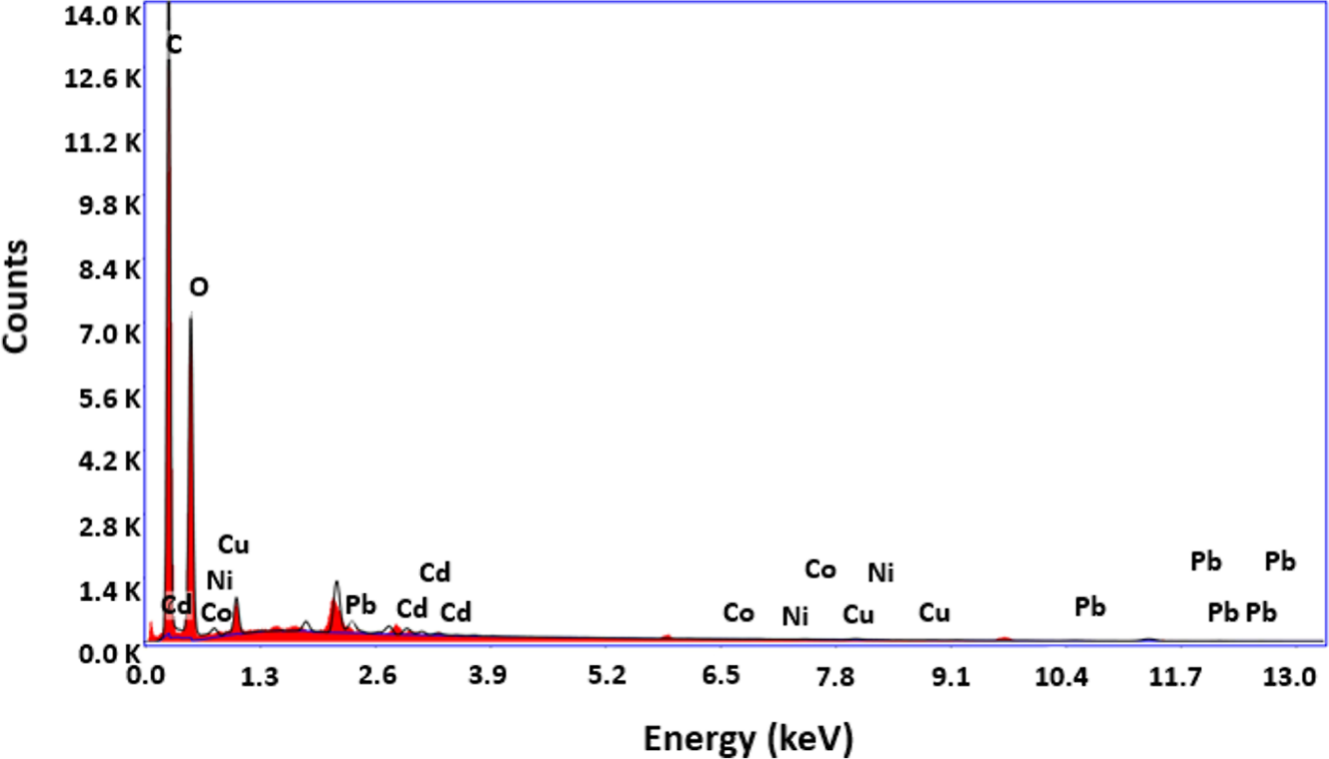
EDS spectrum of hGO after adsorption of heavy metal ions.

**Figure 5:**
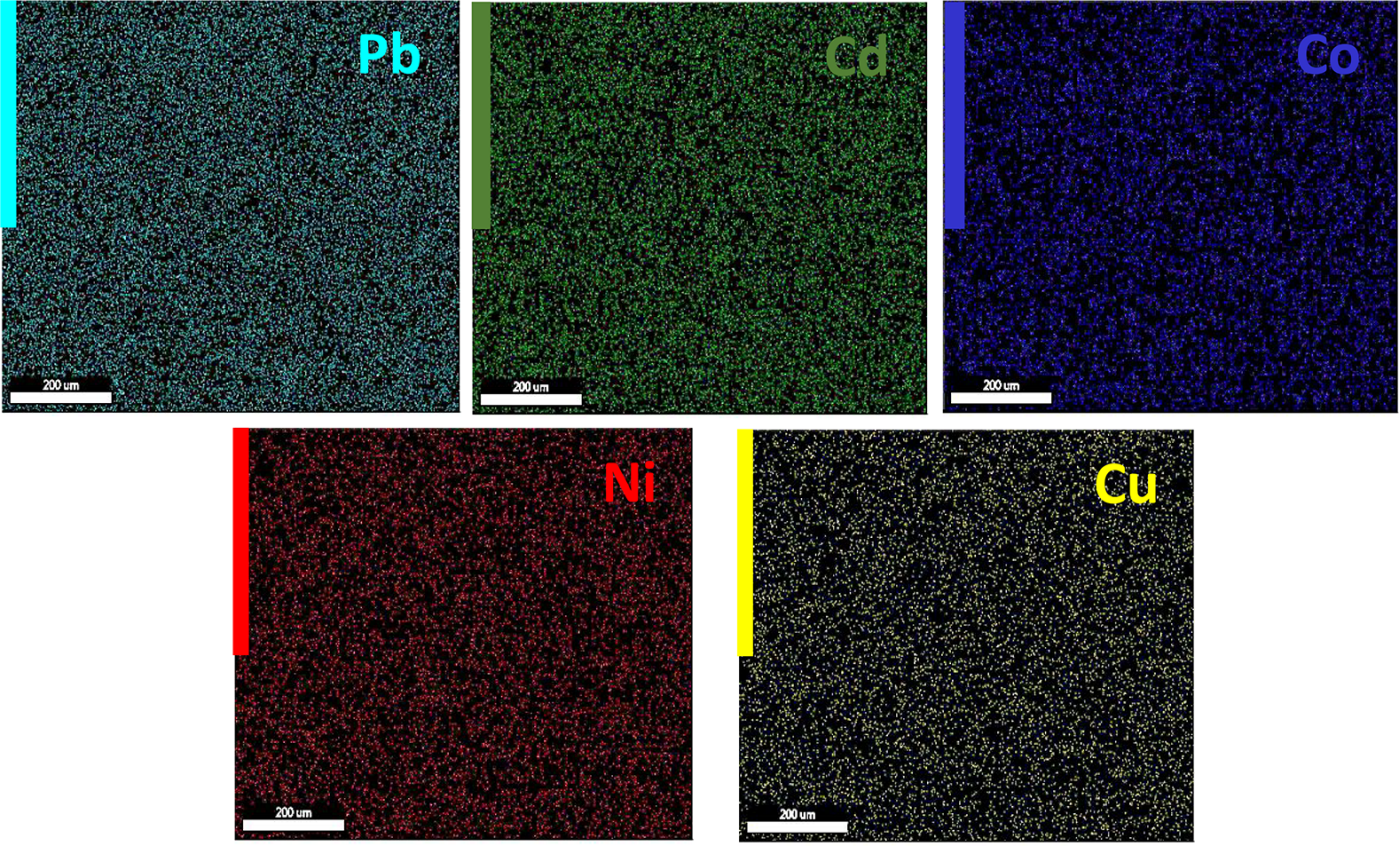
EDS mapping analyses of heavy metal ions adsorbed on hGO.

According to the EDS spectrum of heavy metal ion-adsorbed hGO adsorbent in Figure 4, the characteristic peaks of carbon and oxygen atoms that are the main components of hGO can be seen. On the other hand, the peaks of the Pb (II), Ni (II), Cd (II), Co (II), and Cu (II) metals, indicating the adsorption of heavy metal ions, were successfully achieved on the hGO surface. Further analyses of the adsorption process were carried out by using the EDS mapping technique. The obtained results are given in Figure 5.

The EDS mapping spectra of the hGO surface (after adsorption process) clearly indicate that Pb (II), Ni (II), Cd (II), Co (II), and Cu (II) metal ions were adsorbed by the hGO adsorbent. The dispersion of the adsorbed metal ions was balanced, which can be an evidence of the equal dispersion of the active sites of the hGO adsorbent.^17^

### Optimization of the d-SPE Procedure

The synthesized hGO material was used as an adsorbent for removal of Pb (II), Ni (II), Cd (II), Co (II), and Cu (II) ions in aqueous medium by using the d-SPE technique. This study is the first attempt to use the hGO as an adsorbent in the d-SPE technique according to the literature. Three critical variables (solution pH, adsorbent amount, and ultrasound time) of the d-SPE method were optimized, and the obtained results are given below.

### Effect of Solution pH on the Adsorption

Figure 6 represents the removal efficiency (%) of hGO for heavy metal ions Pb (II), Ni (II), Cd (II), Cr (III), Co (II), and Cu (II) as a function of solution pH, from pH 2 to 8. Results suggested that pH optimization is essential to maximize the heavy metal removal efficiency.

**Figure 6:**
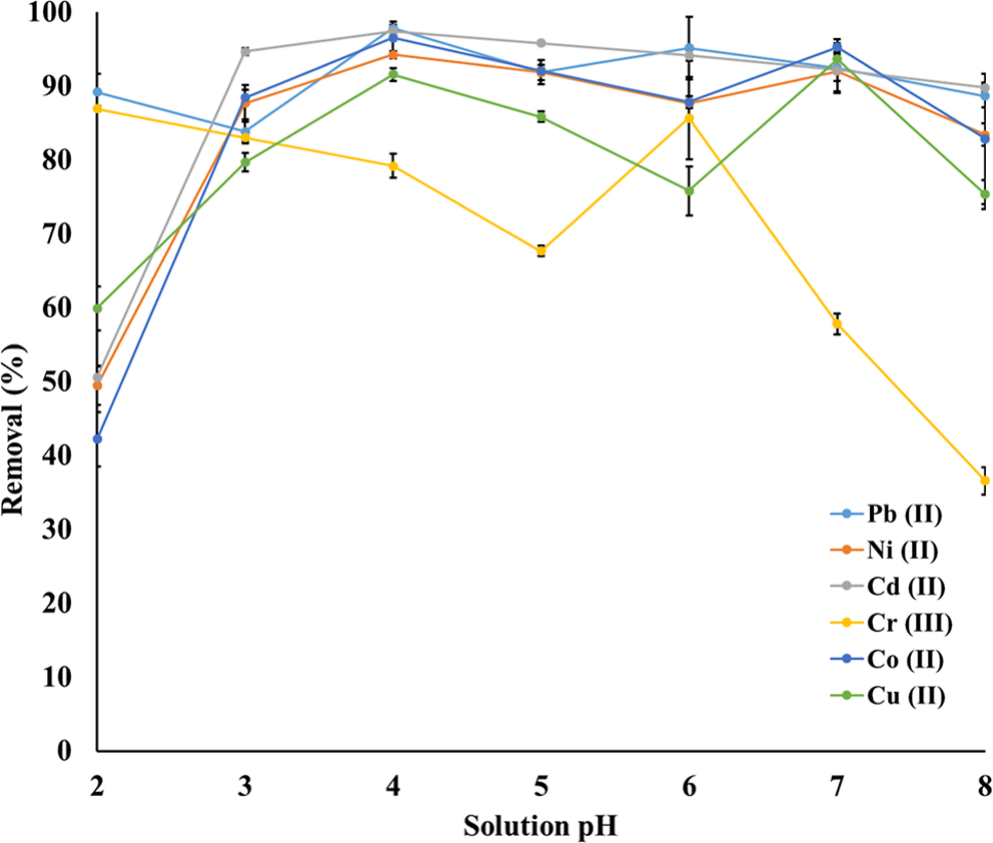
Effect of solution pH on the recovery of Pb (II), Ni (II), Cd (II), Co (II), and Cu (II) ions (*N* = 3).

According to Figure 6, different metal ions exhibit distinct optimum pH and/or pH ranges for optimum removal on the hGO adsorbent. As is seen, Pb (II), Cd (II), and Ni (II) metal ions exhibited higher removal efficiencies across a broad pH range, raising around pH 4–6, whereas Cr (III) showed a significant decrease in removal efficiency as solution pH increased.

Figure 6 clearly indicates that the solution pH significantly affected the removal efficiency of the hGO adsorbent. This tendency can be attributed to the pH influencing both the surface charge of the hGO adsorbent and possibly the ionization state of the metal ions. The competition between H^+^ and heavy metal ions on active sites of the hGO adsorbent adsorption sites may reduce the removal efficiency at pH 2 and pH 3. This competition may lessen with the increasing pH and lead to enhanced adsorption of heavy metal ions. Pb (II), Ni (II), and Cd (II) metal ions showed maximum removal efficiency at a broad pH range (between pH 4 and 6), while Cr (III) showed a steep decline in the removal efficiency above pH 6, which may be attributed to the difference in the ionic radii, hydration energies, and selectivity of the hGO adsorbent due to specific interactions between each metal ion and the functional groups of the hGO surface. Consequently, four of heavy metal ions could be removed from aqueous media quantitatively [Pb (II), Ni (II), Cd (II), and Co (II)], while the removal of Cu (II) ions was around 90% and that of Cr (III) was 79% at pH 4. The rest of the experiments were conducted at pH 4 without chromium, whose removal value was lower than 80%.

### Effect of the hGO Amount

The adsorbent amount used in d-SPE experiments is crucial for optimizing the extraction efficiency, as an adequate adsorbent quantity enhances the interaction between the analytes and the adsorbent.^24,25^

In this study, 5 to 100 mg of hGO adsorbent was evaluated under optimum conditions. The obtained results are illustrated in Figure 7.

**Figure 7:**
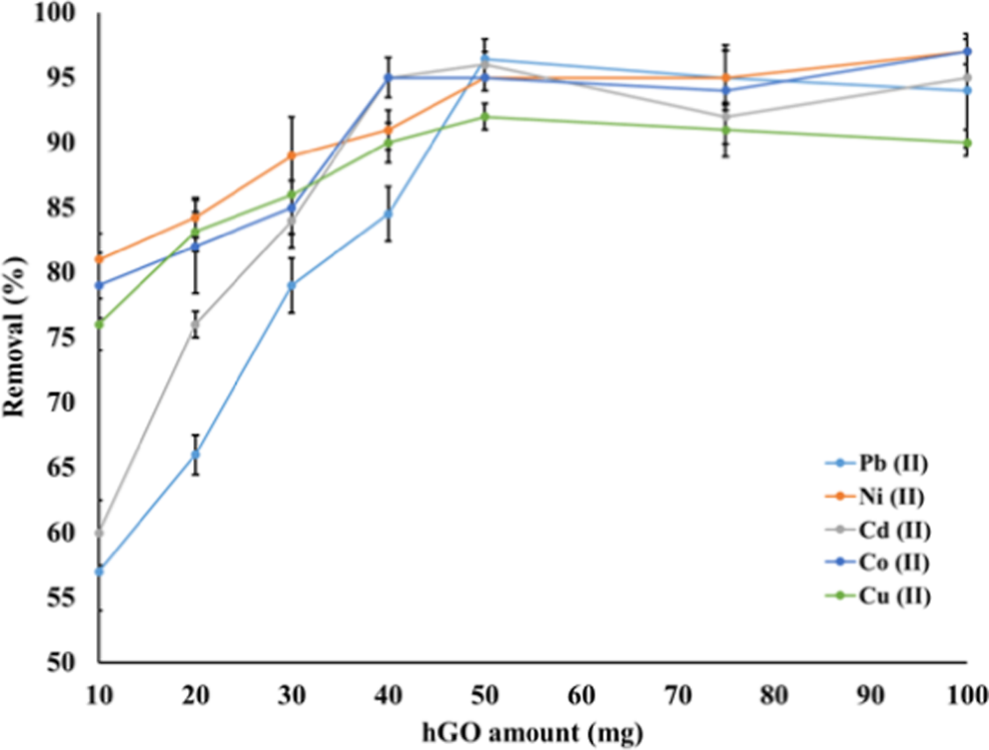
Effect of the hGO adsorbent amount on the d-SPE extraction of Pb (II), Ni (II), Cd (II), Co (II), and Cu (II) ions (*N* = 3).

The data given in Figure 7 demonstrate that the Pb (II) adsorption was particularly efficient, but the hGO adsorbent was also effective in the removal of most metal ions, with removal efficiencies exceeding 95% even at relatively low hGO amounts. The removal efficiency values were raised up to 50 mg adsorbent amount, and beyond this value, the removal values stood stable, which indicates that the maximum capacity of the hGO adsorbent was reached. Consequently, 50 mg of hGO was chosen as optimum for the d-SPE procedure.

### Effect of Ultrasound Time

The ultrasound reduces the operation time and reduces the overall time in the adsorption process between the analyte and adsorbent.^26^ For this purpose, this parameter was studied between 60 and 600 s, and the obtained results are given in Figure 8.

**Figure 8:**
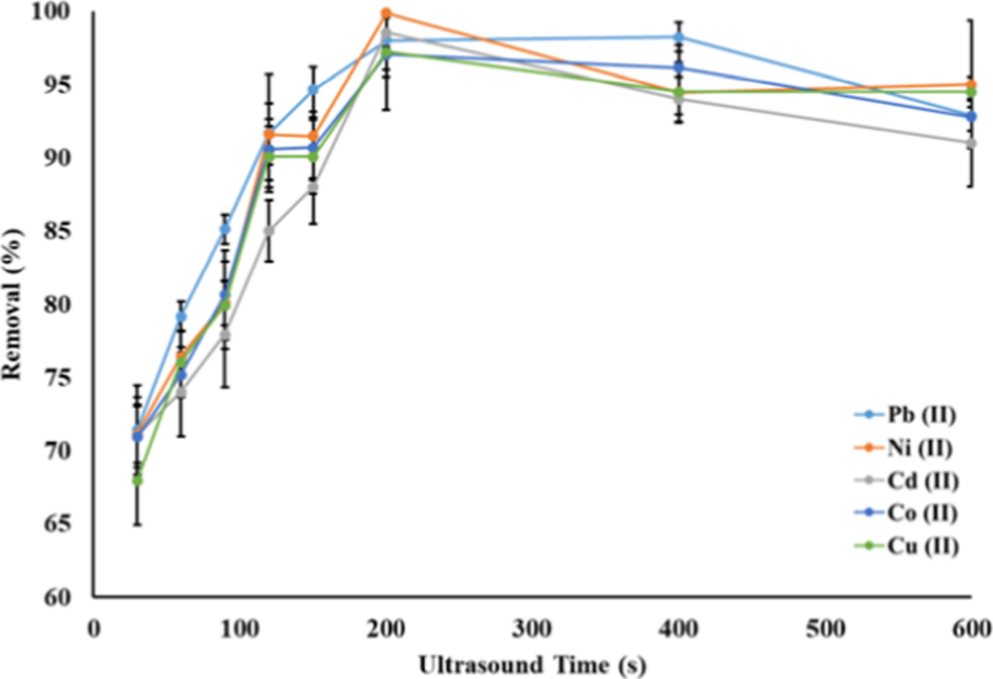
Effect of ultrasound time on the recovery of Pb (II), Ni (II), Cd (II), Co (II), and Cu (II) ions (*N* = 3).

Figure 8 shows the removal efficiency of the hGO adsorbent for the metal ions Pb (II), Ni (II), Cd (II), Co (II), and Cu (II) over ultrasound time. The removal efficiency for all metals showed a rapid increase during ultrasound time. This may be due to the enhanced diffusion and faster interaction between the hGO adsorbent and metal ions by the ultrasound effect. After 200 s of ultrasound application, all metal ions reached an equilibrium to give maximum removal and stood constant after this value. So, 200 s of equilibrium time was chosen as optimum.

### Effect of the Interfering Ions

The influence of potential matrix constituents present in water was systematically investigated to evaluate their interference in the optimized dispersive solid-phase extraction (d-SPE) procedure for the removal of Pb (II), Ni (II), Cd (II), Co (II), and Cu (II) ions. To achieve this, the commonly encountered coexisting ions were introduced into model solutions containing the target analytes and subsequently processed in accordance with the optimized method. The interference criterion was defined based on a decline in quantitative extraction efficiency and standard deviation values exceeding ±5%. The corresponding results are presented in Table 1.

**Table 1:** Effect of Interfering Ions (*N* = 3)^a^

			removal %				
ion	added as	conc. (mg/L)	Pb (II)	Ni (II)	Cd (II)	Co (II)	Cu (II)
Al^3+^	Al(NO_3_)_3_·9H_2_O	25	96 ± 2	94 ± 1	97 ± 3	93 ± 2	89 ± 1
Mn^2+^	Mn(NO_3_)_2_·4H_2_O	25	97 ± 2	95 ± 2	96 ± 3	95 ± 1	90 ± 2
Bi^3+^	Bi(NO_3_)_3_·5H_2_O	25	97 ± 3	95 ± 2	95 ± 3	94 ± 2	90 ± 3
Ca^2+^	Ca(NO_3_)_2_	50	95 ± 2	94 ± 3	95 ± 3	95 ± 2	91 ± 2
Mg^2+^	Mg(NO_3_)_2_·6H_2_O	100	96 ± 3	96 ± 4	98 ± 2	95 ± 3	90 ± 4
Na^+^	NaNO_3_	1000	97 ± 3	95 ± 3	99 ± 4	96 ± 4	91 ± 2
K^+^	KNO_3_	1000	95 ± 2	96 ± 2	98 ± 5	95 ± 3	90 ± 4
NO_3_^–^	NaNO_3_	1000	97 ± 2	97 ± 4	99 ± 5	98 ± 3	92 ± 2
Cl^–^	NaCl	1000	98 ± 3	95 ± 4	98 ± 3	96 ± 3	91 ± 3
PO_4_^3–^	Na_3_PO_4_·12H_2_O	100	97 ± 4	94 ± 2	97 ± 3	95 ± 1	90 ± 1
SO_4_^2–^	Na_2_SO_4_	100	96 ± 3	96 ± 3	97 ± 2	95 ± 4	89 ± 3

a Mean ± standard deviation.

A critical assessment of Table 1 indicates that the investigated interfering ions exhibit negligible detrimental effects on the extraction performance of the target analytes at the specified concentrations under optimal conditions. This observation can be ascribed to the preferential affinity and stronger interactions between the analyte ions and the adsorbent compared to those of the interfering species.

### Adsorption Characteristics

Adsorption characteristics between the hGO adsorbent and metal ions have been investigated in terms of pH_PZC_, adsorption isotherms, and reusability.

### Determination of the Point of Zero Charge (pH_PZC_)

The pH_PZC_ of the adsorbent surface was determined using the mass titration technique. For this purpose, 50 mL of 0.01 M NaCl solution and 100 mg of adsorbent were added in a 250 mL Erlenmeyer. The initial pH values were adjusted from 2 to 9 by using 0.05 M NaOH and 0.05 M HCl. The obtained suspension was stirred at 200 rpm for 48 h. Thereafter, the final pH values were measured by a pH meter after filtration. The pH_PZC_ value of the adsorbent was determined from Figure 9.

**Figure 9:**
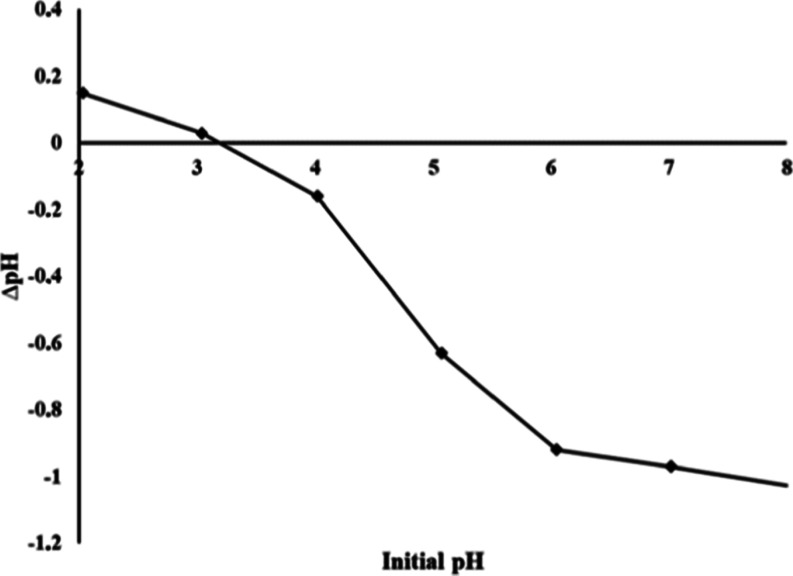
Determination of the net surface charge of the hGO adsorbent.

Figure 9 indicates that the pH_PZC_ value of the hGO adsorbent is 3.23, while the optimum solution pH for the adsorption of Pb (II), Ni (II), Cd (II), Co (II), and Cu (II) ions was pH 4 and above (Figure 6). The point of zero charge (pH_PZC_) is defined as the pH at which the net surface charge of the adsorbent is zero, implying an equilibrium between surface-associated positive and negative charges under fixed conditions.^27^ When the solution pH exceeds the pH_PZC_, the adsorbent surface acquires a net negative charge, whereas at pH values below the pH_PZC_, the surface becomes predominantly positively charged.^28^ Consequently, at the optimal adsorption pH (pH 4), the surface of the hGO adsorbent exhibits a negative charge (optimum pH > pH_PZC_), thereby facilitating electrostatic interactions with cationic species.

The pH_PZC_ is a crucial parameter to explain adsorption behavior and serves as a key indicator of surface interactions. In the present study, the adsorption of Pb (II), Ni (II), Cd (II), Co (II), and Cu (II) ions onto the negatively charged hGO adsorbent is primarily driven by Coulombic forces and electrostatic attractions reinforcing the efficacy of the adsorbent at pH 4.

### Adsorption Isotherm

The adsorption behavior of heavy metal ions on hGO was investigated under optimized conditions, and the results are given in Table 2.

**Table 2:** Parameter Values Fitted from Langmuir Adsorption Isotherm (*T* = 292.15 K, pH = 4, hGO = 50 mg, *V* = 50 mL)

heavy metal ion	*q*_e_ (mg/g)	*q*_max_ (mg/g)	*K*_L_ (L/mg)	*R* ^2^
Pb (II)	2.9492	3.4892	0.3498	0.996
Ni (II)	2.6808	3.0842	0.4030	0.999
Cd (II)	3.156	3.5147	0.5063	0.994
Co (II)	2.7438	3.5455	0.2184	0.994
Cu (II)	2.7762	3.3698	0.2984	0.997

The results from Table 2 revealed that the Langmuir model exhibited excellent fit for all metal ions (*R* ^2^ > 0.99), indicating monolayer adsorption on the adsorbent surface.

The reusability of the hGO adsorbent was analyzed by adsorption–desorption experiments where the heavy metal ions desorbed from the hGO surface by using 0.10 M HNO_3_ in ethanol. The results exhibited that the hGO adsorbent could successfully be used up to 8 times without any performance loss.

The adsorbent used in this study, hGO, is a promising material for heavy metal removal. Table 3 represents a brief comparison of hGO and other adsorbents in the literature.

**Table 3:** Comparison of hGO with the Other Advanced Adsorbents

adsorbent	method	metal ions	removal %	reference
MoS_2_–NFs/SA/PVA	adsorption	Cu (II)	90	^29^
GO/CMC/CS	batch adsorption	Co (II), Mn (II), Cd (II)	43.55, 57.78, 91.38	^30^
4PEI-GO	batch adsorption	Cr (VI)	96.35	^31^
CS-GO	batch adsorption	As (III), As (V)	80, 82	^32^
Fe_3_O_4_@*Pinus pinea*	d-SPE	Cd (II), Co (II), Ni (II)	97, 95, 96	^17^
Fe_3_O_4_@TATS@ATA	batch adsorption	Pb (II)	90	^33^
BNT-APTMS	batch adsorption	Fe (II)	98.9	^34^
hGO	d-SPE	Pb (II), Cd (II), Ni (II), Co (II), Cu (II)	96, 96, 95, 93, 91	present study

### Application on CRM and Real Samples

The applicability, accuracy, and the validation of the proposed d-SPE technique were assessed using a certified standard reference material UME CRM 1204 (elements in wastewater). Real water samples, including natural spring water and industrial process water, were also analyzed to evaluate the performance of the proposed d-SPE method. The obtained results are presented in Tables 4 and 5, respectively.

**Table 4:** Removal of Cd (II), Co (II), Ni (II), and Cu (II) in CRM (*N* = 3)

certified material elements in wastewater UME CRM 1204	certified value (μg L^–1^)	added (μg L^–1^)	removal (%)
Cd (II)	104.5	209.0	^a^96 ± 3
Co (II)	419.0	838.0	95 ± 2
Ni (II)	173.0	346.0	94 ± 3
Cu (II)	334.0	668	91 ± 2

a Mean ± standard deviation.

**Table 5:** Removal of Cd (II), Co (II), Ni (II), Pb (II), and Cu (II) in Real Water Samples (*N* = 3)^a^

	Cd (II)		Co (II)		Ni (II)		Pb (II)		Cu (II)	
real samples	added (μg)	removal (%)	added (μg)	removal (%)	added (μg)	removal (%)	added (μg)	removal (%)	added (μg)	removal (%)
natural spring water		N.D.		N.D		N.D.		N.D.		N.D.
	5.0	97 ± 4	10.0	96 ± 3	10.0	93 ± 4	10.0	97 ± 4	10.0	91 ± 2
factory process wastewater		N.D		N.D						
	5.0	98 ± 3	10	100 ± 5	10.0	94 ± 2	10.0	99 ± 5	10.0	93 ± 4

a Mean ± standard deviation, N.D: not detected.

As presented in Tables 4 and 5, the proposed d-SPE procedure was validated through the precise removal of Cd (II), Co (II), Ni (II), Pb (II), and Cu (II) in certified reference material (CRM) and real samples, including natural spring water and industrial process water.

## Conclusions

In this study, a new nanomaterial, hGO, was synthesized, and its structure was characterized using FTIR, XRD, FESEM, EDS, EDS mapping, and BET techniques. Characterization studies revealed that hGO has a very porous surface structure. The optimization studies showed that Pb (II), Ni (II), Cd (II), Co (II), and Cu (II) heavy metal ions in aqueous solutions were simultaneously and quantitatively removed on hGO by using the d-SPE method. 50 mg of hGO adsorbent was sufficient for the adsorption of heavy metal ions at pH 4 in relatively short equilibrium time of 200 s. This method was successfully applied to real water samples to remove heavy metal ions.
